# Origin, Course, and Angulation of Coronary Artery Anomaly - A Case Report

**DOI:** 10.7759/cureus.28669

**Published:** 2022-09-01

**Authors:** Matheus Roberto Schetz Alves, Júlia Momoli, Emily Lindsey Pilato, Gustavo Lenci Marques

**Affiliations:** 1 Medicine, Pontifical Catholic University of Paraná, Curitiba, BRA; 2 Internal Medicine, Federal University of Parana, Curitiba, BRA

**Keywords:** anomalous coronary artery, anomalous coronary artery origin, coronary circulation, myocardial infarction, coronary vessels, congenital anomalies of coronary arteries

## Abstract

Congenital coronary artery anomalies are a rare diagnosis that can be silent when the patient is asymptomatic. Although these abnormalities may, in most cases, not present clinical alterations, in some cases, they prove to be a cause of myocardial ischemia and sudden death. We report the case of a 20-year-old asymptomatic patient, seen in a routine cardiology consultation, evidenced in an ergometric test ST-segment depression. In this case, follow-up was carried out with coronary angiotomography and scintigraphy to understand the reason for this finding. After the angiotomography has evidenced the diagnosis of anomalous origin and course of the right coronary artery and the trunk of the left coronary artery, in addition to anomalous angulation of the right coronary vessel.

## Introduction

Congenital coronary artery anomalies (CCAA) are, in most cases, incidental findings, either during coronary computed tomography angiography or during an autopsy, presenting a diagnostic challenge as they require a high level of clinical suspicion [1]. Although they commonly present as an isolated finding, without other clinical and/or hemodynamic impacts, silently, these anomalies can have life-threatening repercussions, such as syncope, acute myocardial infarction, and sudden death [2]. However, according to literature, approximately only 20% of cases of anomalous coronary arteries are life-threatening [3]. Even though CCAA represents the second leading cause of death in young athletes (12% of sudden cardiac deaths), often after rigorous physical exercise, and 30% of sudden death cases in young people overall [1-4]. Although they are more evident as an isolated anomaly, it is possible to observe the association of CCAA with heart defects, such as valvular heart disease (bicuspid aortic valve) and congenital heart disease (tetralogy of Fallot or transposition of the great vessels) [3,4]. Regarding prevalence, anomalous coronary arteries are present in approximately 1.5-2% of the general population, with a possible variation between 0.2% and 5.64% found in literature [1,3,5].

The presence of anomalous coronary arteries, as explained above, is a difficult and rare diagnosis and may increase in rarity according to their classification and involvement of one or more of these vessels [1,2]. In this case report, the authors describe the condition of a young and asymptomatic patient without comorbidities who was diagnosed with bilateral anomalous coronary arteries in terms of coronary artery angulation after ECG and routine ergometric test with signs suggestive of myocardial ischemia.

## Case presentation

A 20-year-old male, non-obese and asymptomatic patient is undergoing routine cardiological evaluation to monitor daily use of beta-blocker for essential tremor. No history of arterial hypertension, diabetes, dyslipidemia, smoking, dyspnea, syncope, weakness, and/or chest pain.

**Figure 1:**
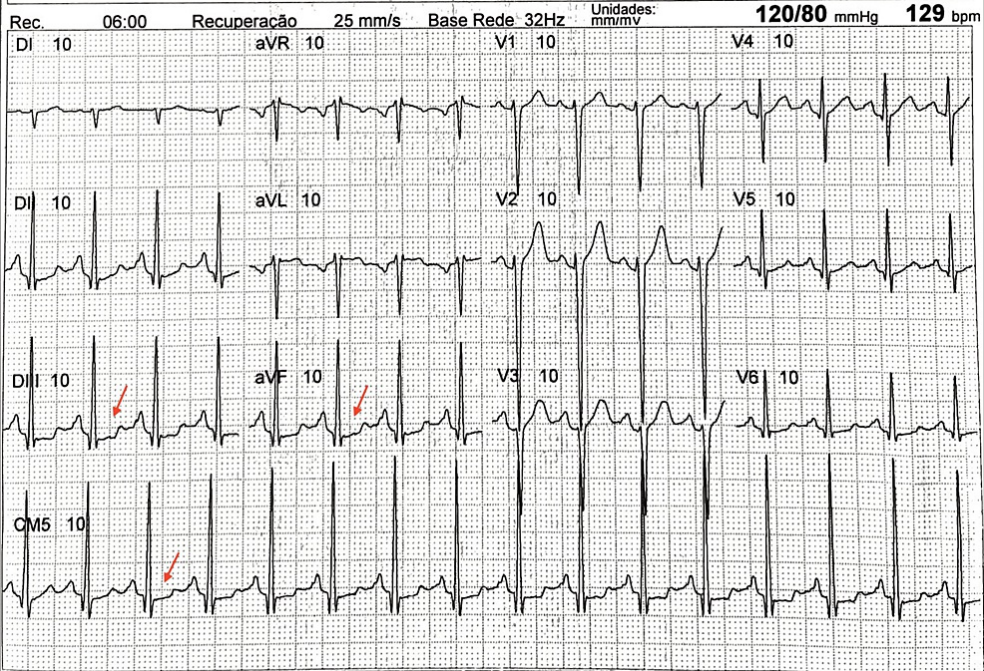
Ergometric test in the final recovery period (06:00) showing an ST depression of 1 mm in the leads DIII, aVF, and CM5

Electrocardiogram and transthoracic echocardiography were performed as initial tests for cardiological follow-up, and both showed all limits within the normal range. Following the evaluation, an ergometric test was performed, which showed a test suggestive of myocardial ischemia in the recovery phase, with horizontal morphology ST-segment depression of up to 1.0 mm in DIII, aVF, and CM5 (Figure 1), without symptoms of coronary insufficiency. It is noteworthy that three years before, the patient had undergone another evaluation with an ergometric test, echocardiogram, and electrocardiogram, in which no change in the ST segment was evidenced.

**Figure 2:**
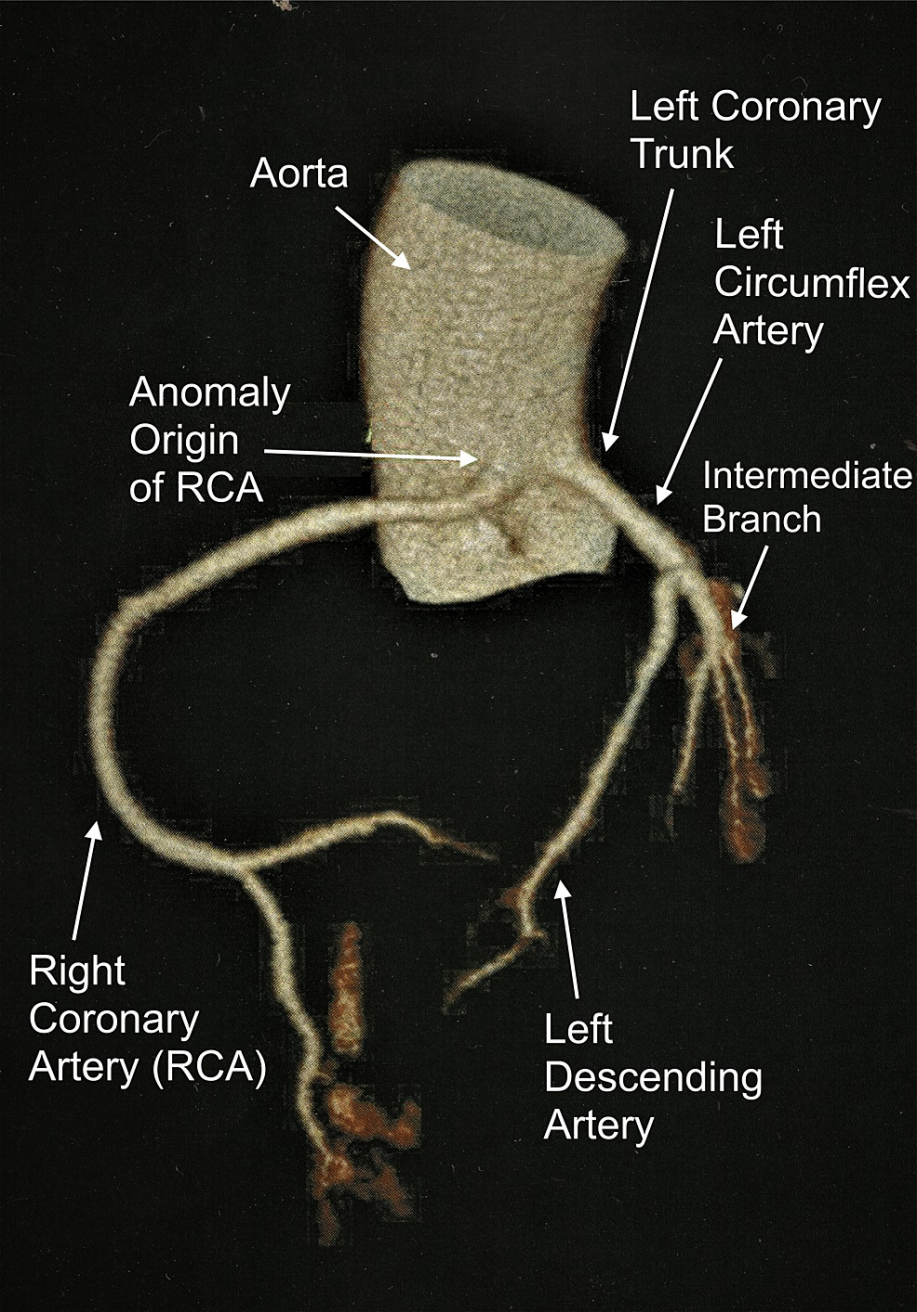
Angiotomography showing the anomalous origin of the right and left coronary arteries RCA - right coronary artery

**Figure 3:**
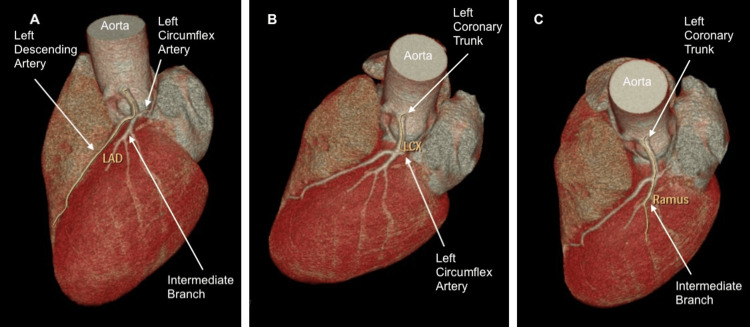
Angiotomography demonstrating the course of the left coronary trunk between the aorta and pulmonary arteries, originating between the left and right coronary sinuses, but just above the sinotubular junction A: Course of the left descending artery; B: Course of the left circumflex artery; C: Course of the intermediate branch LAD - left decending artery, LCX - left circumflex artery

**Figure 4:**
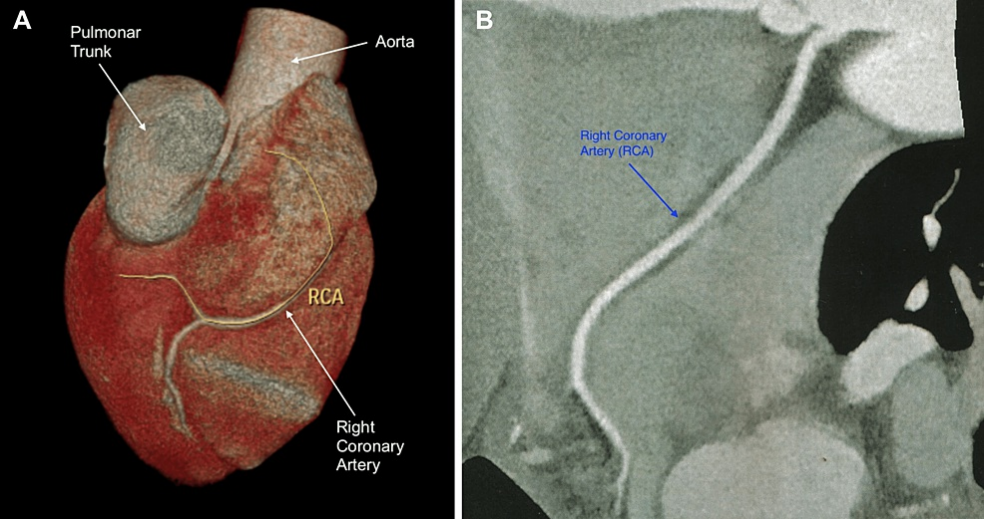
A: Myocardial computed tomography angiography showing a short course of the right coronary artery, with an ostium between the aorta and the RV outflow tract; B: right coronary artery course RV - right ventricular, RCA - right coronary artery

**Figure 5:**
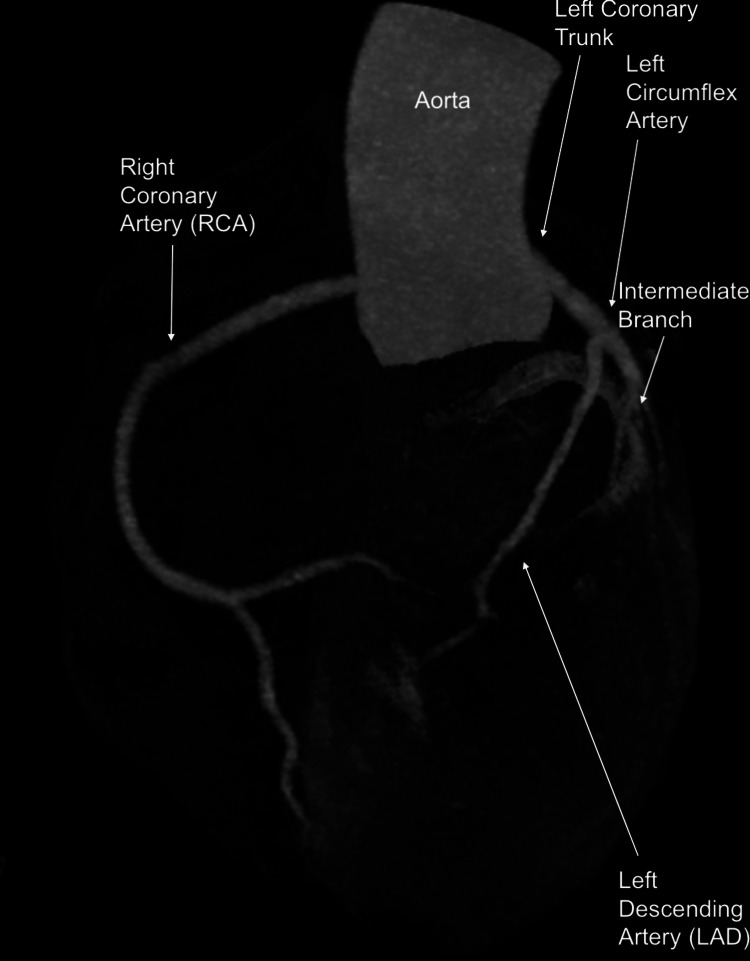
Contrast myocardial tomography angiography demonstrating courses with absence of luminal reduction and/or atherosclerosis

**Figure 6:**
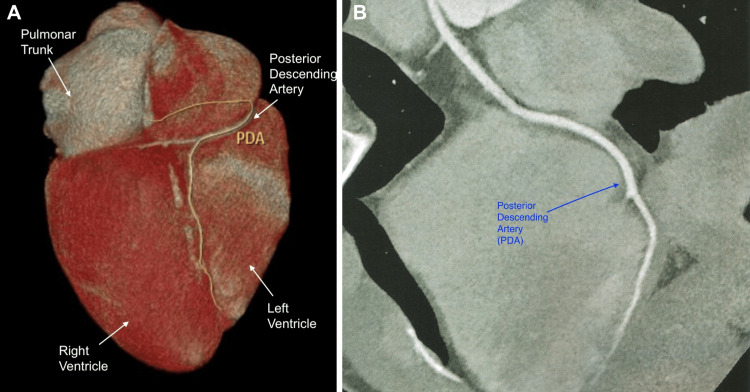
A: Angiotomography showing the PDA course; B: PDA course with no signs of atherosclerosis and luminal reduction PDA - posterior descending artery

To complement the investigation of the ischemic alterations present in the ergometric test, a coronary angiotomography was carried out, and a diagnosis of anomalous origin of the right and left coronary arteries was performed (Figure 2). The left coronary artery trunk originated between the left and right coronary sinuses, but just above the sinotubular junction and with a short course between the aorta and pulmonary arteries, with a rounded and slightly angled ostium, without luminal reduction (Figure 3). The right coronary artery originated between the left and right coronary sinuses, but 3 mm above the sinotubular junction (immediately next to the left coronary ostium), with a short course between the aorta and the right ventricle outflow tract (pulmonary subvalvular) (Figure 4). The ostium of this right vessel was elliptical, and the origin was angled (<45º), with no signs of luminal reduction and atherosclerosis (Figure 5). The posterior descending artery (PDA) branch of the right coronary artery with no signs of atherosclerosis and luminal reduction (Figure 6).

**Figure 7:**
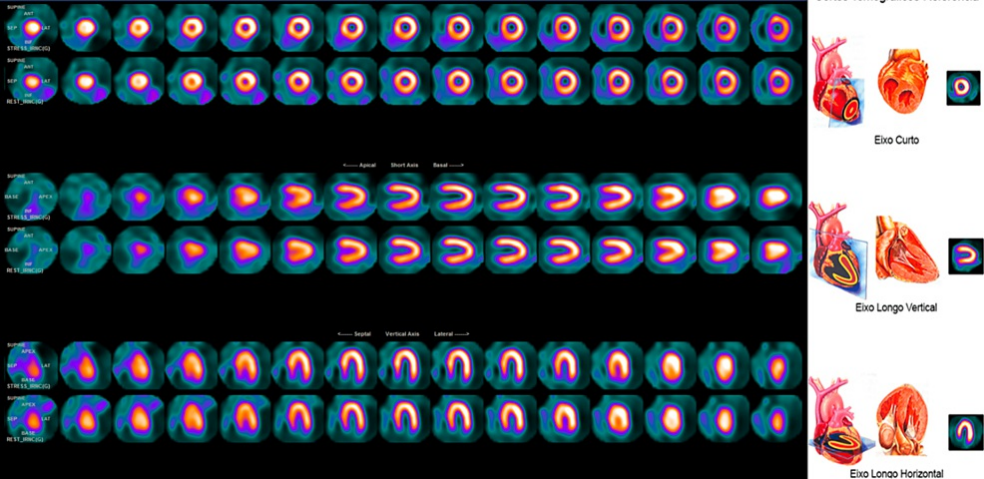
Myocardial scintigraphy showing normal perfusion patterns

Myocardial scintigraphy was performed at rest and after physical exercise with another ergometric test, and it concluded normal myocardial perfusion (Figure 7), normal ventricular function with preserved contractility of the ventricular walls, and preserved left ventricular ejection fraction after exercise. It is noteworthy that, in the ergometric test requested for scintigraphy, there were no manifestations of exercise-induced myocardial ischemia.

Finally, 24-hour Holter monitoring was performed to assess possible changes in the ST segment, and it did not demonstrate significant ST segment changes, only a very rare density of isolated and paired supraventricular extrasystoles.

## Discussion

Anatomy

The anatomy of the coronary arteries, when normal, documents in literature the origin of these vessels as coming from the ostia located in the right and left sinuses of Valsalva, thus arising from the aorta artery and converging to the apex of the heart. There are normally three main coronary vessels: right coronary artery (RCA), left circumflex artery (LCX), and left anterior artery (LAD), the last two originating from the trunk of the left coronary artery (LCA) [2,6,7].

In patients without anomalies, the right coronary artery varies from 12-14 cm in length and originates from the right sinus of Valsalva of the ascending aorta artery, with a course between the right atrium and the pulmonary artery, through the atrioventricular (AV) groove, designating branches along the way. The right coronary artery is responsible for the irrigation of the right atrium, right ventricle, sinoatrial and ventricular nodes, and interatrial septum, in addition to a portion of the left atrium and ventricle [3,6]. Left anterior artery, in general, varies from 10-13 cm in length and originates from the bifurcation of the left coronary artery trunk, with descending course to the left side of the pulmonary artery, through the anterior interventricular sulcus, to curbing itself at the apex of the heart to supply the inferior wall left ventricle. As it develops, LAD gives rise to two groups of vessels: the diagonal branches, which supply the anterior 2/3 of the basal interventricular septum, in addition to the middle and apical part, in its entirety; and the septal perforating branches, which supply the interventricular septum [2,3,6]. Whereas the left circumflex artery is evidenced with a shorter length compared to the other two vessels, having its normal length between 5-8 cm. The left circumflex artery also arises from the bifurcation of the LCA on the left and, during its course, may give rise to the left marginal artery, which presents itself as a large branch [2,3,8]. Finally, LCA is evidenced as originating in the left sinus of Valsalva, crossing between the pulmonary artery and the left atrium, with a short length, because it quickly bifurcates into LAD and LCX [2,8].

Normal variants 

An important point is that, although the coronary vessel does not fully follow the described anatomy, we cannot classify it readily as anomalous since they are presented in normal literature variants that differ from the common anatomy but are still normal. In these cases, we can mention coronary dominance, inferior wall supply, atrioventricular nodal supply, sinoatrial nodal supply, ramus intermedius, right superior septal perforator, supernumerary coronary ostia, myocardial bridging, acute take-off of LCX, high take-off, and Shepherd’s crook RCA [6,8].

The coronary dominance is determined by the artery that supplies the PDA and a posterolateral branch. The artery dominance presents as a normal variant with three different forms: right-dominance (70% of the cases) with supply from RCA, left-dominance (10% of the cases) with supply from LCX, and codominance (20% of the cases). Besides the variability of coronary dominance, the inferior wall supply also presents as a normal variant, represented by early take off of the PDA, multiple branches, and “wraparound LAD”, when LAD supplies the apical inferior wall by wrapping the cardiac apex. Other normal variants are atrioventricular nodal supply, determined by the small branch from the dominant artery that supplies the atrioventricular node, and sinoatrial nodal supply, determined by the vessel that sinoatrial nodal branch arises from RCA (60% of the cases), proximal LCX, distal RCA, or LCX artery. In the ramus intermedius case, this vessel supplies the lateral and inferior walls, and happens when the left main coronary artery (LMCA) trifurcates into a LAD, an LCX, and ramus intermedius. The right superior septal perforator case is the possibility of this vessel supplying the LAD artery with LCA or proximal LAD [6,8].

Another normal variant is the supernumerary coronary ostia, represented by smaller branches rising from the aorta instead of from coronary arteries, exemplified by a separate origin of the conus branch (the most common origin is this vessel arising from proximal RCA) and origins of the LAD and LCX with no common LMCA. The coronary arteries normally travel surrounded by the epicardial fat along the heart, but when this vessel “dives” into the fat, as an atypical intramyocardial course, this represents myocardial bridging. In this case, myocardial ischemia and angina may be found by systolic compression. In a case of acute take-off of LCX, the acute angle of coronary vessel origin (≤ 45º) between LMCA and LCX is the factor in this variant. The high take-off is defined by the position of the coronary ostium and occurs when the ostium is 5 mm or more above the aortic sinotibular junction. Finally, Sheperd's crook RCA is represented by a normal origin of RCA, but a tortuous and high course after origin from the aorta is evidenced [6,8].

Classification of coronary anomalies

The classification of congenital coronary artery anomalies can be performed in several different ways, using references to the normality of the anatomy, as previously mentioned [2,8]. Categorization by comparing anatomical normality, however, can be difficult since any anatomy present in more than 1% of the population is considered normal, which causes a huge variety of possible combinations [9].

One way of categorizing CCAA is according to anatomical-clinical condition, divided into major (abnormalities that cause significant hemodynamic instability, such as myocardial ischemia) and minor (asymptomatic anomalies, which do not cause myocardial ischemia) [3,10]. Currently, however, although there are several classifications, the most common form of organization is the one created by Angelini in 1989, which is constantly being updated. This classification uses anatomical characteristics of the anomalous coronary vessel, dividing them into anomalies of ostium, origin and course, intrinsic and termination [2,3,9]. Other types include congenital absence, hypoplasia, and duplication [2,6].

Anomalies of origin and course include arteries with an anomalously located coronary ostium, those with origin in the opposite coronary sinus (which may follow some courses, such as interarterial, transseptal, pre-pulmonary, and retroaortic), and origin from the aorta or pulmonary artery, in addition to other anomalies, such as a single coronary artery (SCA). The coronary artery anomalies of origin from the aorta are represented by SCA from LSV or RSV; RCA ectopic from RSV, LSV or posterior sinus of Valsalva (PSV); LMCA from PSV; LAD from RCA or RSV; LCX from RSV or RCA. The vessels that originate from the pulmonary artery, RCA, LMCA, LAD, accessory coronary, and other arteries that may be found in these cases arises from the pulmonary artery (PA) [3,6,9,11].

In intrinsic anomalies, arteries with congenital ostial stenosis or atresia (also classified by coronary artery anomalies of ostium), intramyocardial course (myocardial bridge), congenital absence of LMCA, atresia of LMCA, congenital absence of LCX and ectasia or aneurysm are observed. Other types are represented by hypoplasia of RCA and LCX, and anomalies of anatomy evidenced by duplication of RCA, LAD, or LCX [3,6,9,11].

In termination anomalies, congenital fistula of the coronary artery, extracardiac terminations, and systemic termination, when the coronary artery arises into a systemic artery are evidenced [3,6,9,11]. Table 1 shows a classification scheme with all cited classifications, including some groups that are rare in literature [2,6]..

**Table 1:** Classification of coronary anomalies based on normal anatomy PA - pulmonary artery; RCA - right coronary artery; LMCA - left main coronary artery; LAD - left anterior descending; SCA - single coronary artery; LSV - left sinus of Valsalva; RSV - right sinus of Valsalva; PSV - posterior sinus of Valsalva; LCX - left circumflex artery; LCA - left coronary artery; PD - posterior descending branch

Classification of coronary anomalies based on normal anatomy [2,6]			
Anomalies of ostium	Ostial atresia		
	Valve-like ridge		
	Anomalous location of coronary ostium within aortic root or near proper aortic sinus of Valsalva (for each artery)	High	
				Low	
		Commissural			
				Anomalies of origin	From pulmonary artery (PA)	Right coronary artery from PA	
		Left main coronary artery (LMCA) from PA					
		All from PA			
Accessory coronary from PA					
		LCA that arises from posterior facing sinus			
Circumflex that arises from posterior facing sinus					
		LAD that arises from posterior facing sinus			
RCA that arise from anterior right-facing sinus					
		Ectopic location (outside facing sinuses) of cany coronary artery from pulmonary	From anterior left sinus		
From pulmonary trunk					
	From pulmonary branch				
From aorta			Single coronary artery (SCA)			SCA from left sinus of Valsalva (LSV)
	SCA from right sinus of Valsalva (RSV)					
						Right coronary artery (RCA)	RCA ectopic from RSV
	RCA from LSV						
		RCA from posterior sinus of Valsalva (PSV)				
	LMCA		LMCA from PSV			
		Left anerior descending (LAD)					LAD from RCA	
	LAD from RSV							
			Left circumflex artery			LCX from RSV	
	LCX from RCA						
Anomalies of course					Absent left main trunk (split origination of LCA)		
	Anomalous location of coronary ostium outside normal “coronary” aortic sinuses	Right posterior aortic sinus					
					Ascending aorta	
		Left ventricle				
		Pulmonary artery (2.a.)		
		Aortic arch		
		Innominate artery		
		Internal mammary artery		
		Bronchial artery		
		Subclavian artery		
		Descending thoracic aorta		
		Anomalous location of coronary ostium at improper sinus	RCA that arises from the left anterior sinus, with anomalous course		Posterior atrioventricular groove or retrocardiac
						Retroaortic
	Between aorta and pulmonary artery (intramural)					
					Intraseptal
	Anterior to pulmonary outflow				
					Posteroanterior interventricular groove (wraparound)
	LAD that arises from right anterior sinus, with anomalous course					Between aorta and pulmonary artery (intramural)
					Intraseptal	
				Anterior to pulmonary outflow	
					Posteroanterior interventricular groove (wraparound)
				Circumflex artery that arises from right anterior sinus, with anomalous course		Posterior atrioventricular groove
					Retroaortic	
			LCA that arises from the right anterior sinus, with anomalous course			Posterior atrioventricular groove
					Retroaortic	
	Between aorta and pulmonary artery				
				Intraseptal
	Anterior to pulmonary outflow			
				Posteroanterior interventricular groove
	Single coronary artery			
				Anomalies of anatomy	Duplication	Of RCA	
	Of LAD						
	Of LCX				
	Intrinsic coronary arterial anatomy					Congenital ostial stenosis or atresia (LCA, LAD, RCA, LCX)	
					Coronary ostial simple		
			Coronary ectasia or aneurysm			
			Absent coronary artery	
					Coronary hypoplasia	
			Intramural coronary artery (muscular bridge)			
					Subendocardial coronary course	
			Coronary crossing			
					Anomalous origination of posterior descending artery from the anterior descending branch or a septal penetrating branch	
			Split RCA				Proximal + distal posterior descending (PD) that both arise from RCA
					Proximal PD that arises from RCA, distal PD that arises from LAD		
						Parallel PDs x2 (arising from RCA, LCX) or “codominant”
					Split LAD		LAD + first large septal branch
						LAD, double (parallel LADs)	
							Ectopic origination of first septal branch	RCA
			Right sinus					
		Diagonal					
		Ramus					
		LCX					
Anomalies of termination		Fistulas from RCA, LCA or infundibular artery to	Right ventricle				
					Right atrium	
			Coronary sinus	
			Superior vena cava	
			Pulmonary artery	
			Pulmonary vein	
			Left atrium	
			Left ventricle	
			Multiple, right + left ventricles	
			Systemic termination		
		Inadequate arteriolar/capillary ramifications		
					Congenital absence	Congenital absence of LMCA		
		Atresia of LMCA						
Congenital absence of LCX					
Hypoplasia	Hypoplasia of RCA and LCX				
Anomalous anastomotic vessels			

According to Angelini’s updated classification, the congenital coronary artery anomalies of the patient reported are categorized as an anomaly of origin and course. For this classification, we observed an anomalous origin of the ostia, located between the right and left coronary sinuses, immediately next to each other, and following interarterial course, both for the right coronary artery and the left coronary artery. This course evidenced in the patient is commonly described as a malignant course and is characterized as the origin of the coronary artery in an anomalous form from the opposite coronary sinus, obtaining a course between the aorta and pulmonary artery or between the right ventricular outflow tract [2,3,9,12]. The malignancy of this segment is evidenced by the increased risk of sudden death in patients with this anomaly, being described as present in 80% of autopsies of sudden death in athletes due to coronary anomalies [2,12,13]. It is believed that the higher incidence of sudden death in this anomaly is associated with the mechanism of lateral compression during systole with tachycardia, or increased systolic volume, generating a narrowing of the coronary lumen at the local where it crosses from the anomalous side to the normal one [14,15]. However, the pathophysiology is still controversial and generates disagreement between authors [16].

Symptoms of coronary anomalies

The symptomatology of congenital coronary artery anomalies in most cases is silent; thus, the patient can remain asymptomatic throughout life without any clinical and/or hemodynamic manifestation [2,3,12]. However, approximately 20% of cases are associated, in addition to sudden death and myocardial ischemia, with symptoms such as angina, dyspnea, syncope, cardiomyopathy, and ventricular fibrillation [3,12,17]. Furthermore, clinical consequences other than those strictly correlated to myocardial ischemia are evidence, such as volume overload and aortic root distortion, present in cases of coronary fistulas. Other disorders generated by CCAA also include bacterial endocarditis, misdiagnosis, and complications in aortic valve surgery or angioplasty [11,14].

According to literature, the symptoms generated by CCAA are predominantly associated with athlete patients or after intense physical exercise, being rarely evidenced in sedentary individuals, and may vary according to the classification of the anomalous vessel [2,14]. Coronary arteries with an origin from the opposite coronary sinus and with a malignant course are associated with major clinical consequences, including death, in young athletes and military. In cases of sudden death, in addition to a peak prevalence at 18.5 years of age, there is no high risk in patients with the same defects at older ages. On the other hand, the origin of the left coronary artery from the pulmonary artery is associated with anterolateral myocardial infarction in newborns [14].

Another important point about the symptomatology of anomalous coronary arteries is that, based on studies, the abnormalities previously described do not make the vessels more susceptible to atherosclerotic obstructive disease when compared to normal coronary arteries [18].

Diagnosis

Coronary angiography is the gold standard test for diagnosing coronary artery anomalies through a two-dimensional analysis. However, due to the lack of precision in determining the course of these arteries by this type of analysis, coronary computed tomography angiography (CTA) is a potentially used alternative [9,19].

The CTA is a non-invasive imaging test that allows the control and the investigation of the origin, angulation, relation with other cardiac structures, and course of the anomalous coronary arteries. Besides, in case of surgical need, it can assist in the intervention plan [9,19].

Other tests such as ECG and transthoracic echocardiogram can also be used. However, as most patients are asymptomatic, the presence of ischemia and/or arrhythmias is observed only in isolated cases. In addition, the study of the origin and course of the coronary arteries based on the echocardiogram proves to be the dependent operator for its success and correct analysis, which makes its use for the diagnosis difficult [9,19].

Treatment

Congenital coronary artery anomalies do not yet have a specific treatment method, especially when it comes to asymptomatic cases [16]. However, there is a consensus among experts on surgical intervention to correct these anomalies when the malignant (interarterial) course of these vessels is evident, even without guidelines [12,16]. Treatment with beta-blockers and withdrawal from activities with high physical effort and at a competitive level, are also reported. Besides, surgical interventions can be used in cases of malignant courses and in anomalies generating life-threatening manifestations that make the patient’s autonomy impossible [9,12].

In this case report, as the patient was asymptomatic and without significant ischemia in the provocative test, the chosen conduct was to keep the drug treatment with the use of beta-blockers, even though he was already using it for other purposes. In addition, considering an active patient and the association between interaterial course with sudden death after intense physical exercise, it was recommended to avoid activities that generate high effort. Also, the absence of symptoms associated with this anomaly after continuing drug treatment encourages the option of non-invasive treatment. Approximately one year after diagnosis, with the instituted therapy, the patient remained asymptomatic, without limitations of usual activities, practicing physical exercises normally.

## Conclusions

Congenital coronary artery anomalies (CCAA) are a rare finding, both because of the rarity of anomalies and because, in most cases, they present themselves asymptomatically and with normal tests, manifesting only suddenly, when life-threatening. As they manifest themselves in different ways, and with different anomalous anatomies, CCAA can be divided into some classifications, according to the anatomical-clinical condition. The patient reported was classified with an interarterial course, although it its short and without clinical manifestations. This classification demands notoriety because this course is commonly associated with infarction or sudden death caused by CCAA, being judged as a malignant course. It is concluded, therefore, the need for investigation, mainly with CTA, for cases of young patients with signs of myocardial ischemia, without signs of atherosclerosis, or young athletes in high physical stress. Treatment is variable, with preference for conserved treatments, and must be individualized.
